# Pairing of white wine made with shade-grown grapes and Japanese cuisine

**DOI:** 10.1038/s41538-021-00089-0

**Published:** 2021-03-01

**Authors:** Takuji Takahashi, Kumiko Nakano, Machiko Yamashita, Hanae Yamazaki, Tohru Fushiki

**Affiliations:** 1 Department of Food and Agricultural Science, Graduate School of Agriculture, Ryukoku University, Otsu, Japan; 2 Department of Life Environment, Koshien Junior College, Nishinomiya, Japan; 3 Experimental Farm, Kyoto University, Kyoto, Japan

**Keywords:** Agriculture, Metabolic engineering

## Abstract

This study investigated the effect of pairing of wine vinified from shade-grown grapes before onset of ripening on the palatability of sashimi, a typical Japanese cuisine. GC-MS analyses of volatile chemicals revealed that shading reduced phenolic compounds and terpenoids, and added fatty acid ethyl esters which are also known to contribute to the flavor of Japanese sake. When the pairing of sashimi with wine was evaluated by individuals who regularly drink Japanese sake during meals, shade wine was more highly rated than wine made from normally-grown grapes.

The number of restaurants around the world offering Japanese cuisine is markedly increasing, having reached 118,000 restaurants in 2017 as estimated by the Ministry of Agriculture, Forestry, and Fisheries. Given the increase in foreigners visiting Japan, interest in and appreciation for Japanese cuisine have been on the rise^1^. Within Japan as well, wine consumption has markedly increased in recent years and the demand for wine that pairs well with Japanese cuisine has increased. However, no major effort has been made on research and development to this end.

Grape berries contain various terpene compounds that contribute to a floral flavor, along with other compounds that are formed during fermentation. While a number of cooking experts and sommeliers^2^ have attempted to pair this characteristic flavor with Japanese dishes to bring out the flavor of fish, the development of new types of wine with a focus on grape cultivation methods remains a challenge.

Aromatic components and their precursors in grapes that are involved in forming the aroma of wine are produced at the end of the ripening stage of berry development. In particular, light has a large impact on the production of aromatic components, and shading and blocking UV light has been reported to suppress the production of these components^3,4^. According to a detailed analysis of the components of Chardonnay grapes and Japanese white grapes grown in the shade by Bahena et al.,^5^ reductions as well as changes in the composition of monoterpenoids and aroma-related phenol compounds were observed. The present study evaluated the pairing of Japanese cuisine with an experimental wine (shade wine) vinified from shade-grown Japanese grape berries. The comparator wine was vinified in a similar manner, but used grape berries which were not shade grown (non-shade wine).

We initially had sommeliers in our research group speak an impression of the taste of the shade wine. The sommeliers noted that shading produced a calm taste which was similar in tone to that of Japanese sake brewed from the finest rice. As shown in Table 1, qualitative analysis of aromatic compounds in the wines by GC-MS supported the sommeliers’ impression, in that shading reduced the concentration of vinylguaiacol, terpene-4-ol, alpha terpineol, and ethyl acetate, consistent with Bahena’s report that shading reduces volatile phenolic compounds and terpenoids in berries. Shading also added many fatty acid ethyl esters, which are flavor compounds found in Japanese sake. In particular, 2-hexanoic acid ethyl ester is found in high-quality Japanese sake^6^. Other flavor compounds in shade wine that are also found in matured Japanese sake include diethyl succinate and furfural, which confer a rich aroma of esters from brewing fine rice and restrain the volatile flavor of terpenoids.

**Table 1:** Difference of aroma compounds between shade wine and non-shade wine.

Compounds with increased peak area	Peak area ratio (shade/non shade)
Dodecanoic acid, ethyl ester	7.68
Diethyl succinate	3.39
Furfural	2.65
2-hexenoic acid, ethyl ester	2.53
Isobutyric acid	2.31
Decanoic acid, ethyl ester	2.10
Ethyl hydrogen succinate	1.87
Decanal	1.72
Blackberry thiophenone	1.62
Ethyl isovalerate	1.59

Compound with decreased peak area	Peak area ratio (shade/non shade)
4-Vynylguaiacol	0.018
E)-beta-damascenone	0.045
4-carvomenthenol	0.096
Terpinen-4-ol	0.356
Nonanal	0.360
Methyl salicylate	0.369
Ethyl lactate	0.407
β-phenylethyl butyrate	0.480
Benzene, 1-methyl-3-(1-methylethyl)-	0.485
α-Terpineol	0.531

The peak are ratio corresponds to mean ratio of five measurements each for shade and non-shade wine. Compounds in the Table correspond to those which were statistically significant on the volcano plot (*p* < 0.05).

Ninety-one Japanese women (20–68 years) participated in this study. The impact of habitual differences in alcoholic beverages consumed during meals on the evaluation of pairings cannot be ignored. Accordingly, in addition to analyzing all participants, we conducted sub-analyses by dividing participants into sake drinkers who regularly drink Japanese sake during meals (*n* = 47) and sake non-drinkers (*n* = 44) who typically drink wine or beer during meals. Participants evaluated the overall palatability of the dishes when taken together with wine based on a visual analog scale (VAS). No significant difference was observed between shade wine and non-shade wine in pairings with sashimi in the overall study population (shade wine: 71 ± 18; non-shade wine: 70 ± 20; *n* = 91). However, as shown in Table 2, two-way analysis of variance revealed a significant interaction between the effect of sake drinkers or sake non-drinkers and the effect of shade wine or non-shade wine (*F*(1,89) = 6.364, *p* = 0.0134). Bonferroni’s multiple comparisons test revealed a significant difference between (shade wine with sashimi × regular drinking of Japanese sake during meals) vs. (shade wine with sashimi × not drinking Japanese sake during meals) (*F* (3, 178) = 2.973, *p* = 0.033).

**Table 2:** Difference in palatability of sashimi paired with shade wine or non-shade wine according to sake drinking status.

	Sake drinker *n* = 47			Sake non-drinker *n* = 44			ANOVA	
	*M*	SD	*M*	SD	Effect	*F* ratio	*p* values	partial *η*^2^
Shade wine with sashimi	77.8	16.9	66.0	22.4	D	0.43	0.514	0.70
Non-shade wine with sashimi	69.3	20.3	71.0	18.4	W	2.62	0.109	0.08

*ANOVA* Two-way analysis of variance, *M* Means, *SD* Standard deviation.

D = Sake drinker/sake non-drinker, W = Shade wine/non-shade wine.

No significant difference of VAS was observed between shade wine with sashimi × not drinking Japanese sake during meals vs. non-shade wine with sashimi × not drinking Japanese sake during meals, suggesting that regular drinking of Japanese sake during meals contributed to the increased palatability of raw fish paired with shade wine. Meanwhile, the palatability of steamed vegetables was not influenced by the method of cultivation or experience with sake drinking (data not shown). These results suggest that shade wine may pair remarkably well with Japanese cuisine, in particular, to bring out the flavor of raw fish.

We also analyzed the influence of the taste of raw fish on the overall evaluation of the pairings based on prior experience with drinking Japanese sake^7^. Eight questions were selected based on established methods for assessing the comprehensive palatability of cheese and Japanese sake, with a focus on three aspects related to reward, culture, and information. Significantly higher evaluations by sake drinkers were observed only for the cultural aspe1cts of comprehensive palatability, as reflected in comments such as “I am used to this taste,” and “I have seen this food in advertisements or heard of it before” (Supplementary Table S1). In other words, for sake drinkers, a sense of familiarity had the greatest impact on the quality of the pairing of sashimi with white wine.

Sashimi such as the photograph in Fig. 1 with soy sauce is a characteristic and popular Japanese dish that places weight on enjoying the fresh taste and aroma unique to each fish. While many views exist regarding pairings with wine, there are reports that sake drinkers prefer pairings that allow one to enjoy the taste of raw fish and its gentle fading away upon drinking sake^2,8^. Many participants of the present study reported views that shade wine is an “alcoholic beverage that brings out the delicate taste of ingredients,” reflecting the high expectations for this type of wine. In the development of wine that pairs well with Japanese cuisine, it will be important to consider not only the simple matching of taste and smell, but also differences in the perspective of taste.

**Fig. 1:**
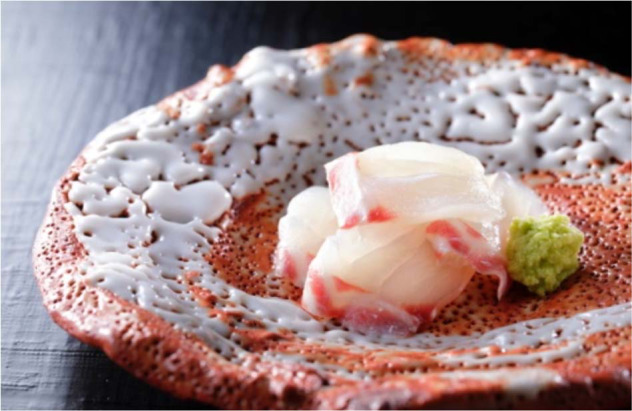
Red sea bream sashimi. Red sea bream is long been favored in Japan for its appearance and flavor. Sashimi is fish sliced thinly. Cooked and photographed by T. Takahashi.

Although there is a need to further refine shade wine, our findings provide a technical basis for designing wines that pair well with Japanese cuisine, i.e., by controlling exposure to light when growing grapes.

## Methods

A total of 91 Japanese women (age range, 20–68 years) consented to participate in this study, which was conducted in accordance with the principles set forth in the Declaration of Helsinki. This study was approved by the Ryukoku University Ethics Committee on Human Studies (Approval No. 2015-04).

The wine and Japanese cuisine pairing evaluations were conducted in a Japanese-style tatami room of a restaurant. The temperature of the room was set to 25 °C. The wines used in the study were stored in a refrigerator at 10 °C until just before the evaluations and were presented to participants such that they were blinded to the type of wine being offered. Participants were asked to rate the palatability of the dishes when wine filled the mouth. Overall palatability was rated on a 100 mm straight-line visual analog scale^9^ (VAS; 0 mm = not delicious at all, 100 mm = very delicious).

Further analyzes of pairings of wine and dishes were performed to evaluate the contribution of three subdomains considered to constitute overall palatability (reward, culture, and information)^8^. For this purpose, a questionnaire was prepared with 8 questions (Supplementary Table S1), which were answered using a 5-point Likert scale (1 = none at all; 5 = very much so). Prism 8 (Graph pad) was used for statistical analyses. Other detailed methods are available as Supplemental Information.

## Supplementary information

Supplemental Information

## Data Availability

Data supporting the findings reported herein are available on reasonable request from the corresponding author.
