# Supplementary material for: Pairing of white wine made with shade-grown grapes and Japanese cuisine
Source: NPJ Sci Food. 2021 Mar 1;5:5. doi: 10.1038/s41538-021-00089-0 (PMC7921643; doi:10.1038/s41538-021-00089-0)
Supplement: Supplementary file 1 — Supplemental Information [file 41538_2021_89_MOESM1_ESM.pdf]

Supplemental Information

Pairing of white wine made with shade-grown grapes and Japanese cuisine

Authors: Takuji Takahashi[1], Kumiko Nakano[2], Machiko Yamashita[3], Hanae Yamazaki[1], Tohru Fushiki[1]

1) Department of Food and Agricultural Science, Graduate School of Agriculture, Ryukoku University, Japan
2) Department of Life Environment, Koshien Junior College, Japan
3) Experimental Farm, Kyoto University, Japan

Supplemental Methods

Cultivation of grapes

Domestic grape berries were covered with a paper-based shading bag (290 x 200 mm, black inner surface) to block both light and UV light starting two weeks before veraison (onset of ripening). Grapes (3.5 tons) were harvested from a vineyard (4,200 m^2) to make 2,300 L of fruit juice, which yielded a total of 2,600 bottles (740 mL/bottle) of shade wine. The comparator wine (non-shade wine) was made by the same wine maker under the same conditions as shade wine, except that light and UV light were not blocked. These wines were provided by a winemaker in Yamanashi prefecture. Sashimi and steamed vegetables were made at a restaurant immediately before evaluation by participants.

Coding of wine

To differentiate between the two types of wine during the study period, shade wine was coded as 207 and the non-shade wine as 306.

Preparation of dishes

Two traditional Japanese dishes were prepared. The first dish was sea bream sashimi, which was simply rinsed in fresh water before being sliced. The second (control) dish was steamed vegetables (stewed turnips). Sashimi and steamed vegetables were made in the kitchen of the restaurant where the study took place immediately before the evaluations.

Test methods

The impact of habitual differences in alcoholic beverages consumed during meals on the evaluation of pairings cannot ignored. Accordingly, in addition to analyzing all 91 participants, we conducted sub-analyses by dividing participants into Japanese sake drinkers (N=47) and sake non-drinkers (n=44). Sake non-drinkers mainly drink wine or beer during meals. The room temperature was set to 25ºC. The wines were stored in a refrigerator at 10ºC until just before the evaluations and were presented only as code numbers to participants to blind them to the type of wine being offered.

Test design

Experiments were carried out so as to replicate an actual dinner setting. Participants were divided into 3 groups, and the experiment was performed for 3 consecutive days in the same manner. After explaining the test procedure, wine 207 (shade wine), wine 306 (non-shade wine), and water in a wine glass, together with 3 pieces of sashimi on a plate, were served. Wine glasses were placed on a designated spot (circle marked with the number 207, 306, or water) on the table.

Participants were first asked to dip one piece of sashimi in soy sauce and place it in the mouth. After chewing 2 or 3 times, participants filled their mouths with wine 207 before swallowing the sashimi. The total palatability of sashimi when wine filled the mouth was then rated on a 100 mm straight-line visual analog scale (VAS). After this VAS rating, participants were asked to answer 8 questions using a 5-point Likert scale. Following the test with wine 207, participants were asked to go through the same steps with wine 306. After completion, participants provided free comments about the pairing of the dish and wine.

Following the sashimi evaluation, steamed vegetables were also evaluated using the same procedure. Prior to this evaluation, the order of wines 207 and 306 were switched. The order of wine tasting was also changed every day. However, the order of sashimi and steamed vegetables was not changed.

VAS-based evaluation of comprehensive palatability of sashimi with wine

VAS was used to evaluate the comprehensive palatability of sashimi in accordance with the method described by Prescott (2004). The use of an unstructured or linear hedonic scale has the advantage of giving judges more freedom to express their sensory perceptions. We used the term “palatability” in its colloquial sense that reflects a positive hedonic evaluation under a given set of conditions. A system for analyzing the pairing of raw fish with wine has not yet been established. Accordingly, we investigated various factors related to the compatibility of raw fish and wine.

Data analysis

Two-way analysis of variance was used to analyze experimental data. Given the presence of interactions, we used Bonferroni’s multiple comparisons test to compare all groups.

GC-MS analysis

Conditions for GC-MS analyses were as follows:

GC-MS: GCMS QP-2010 Ultra (Shimadzu)

Column: DB WAX 60 × 0.25 mm, 0.25 mm (Agilent)

SPME fiber: 50/30 mm, DVB/CAR/PDMS, fused silica, 24 Ga (Supelco)

Auto sampler: AOC5000 plus (Shimadzu)

Oven: 3 min hold at 40ºC → 5ºC/min to 110ºC → 10ºC/min to 240ºC → 5 min hold at 240ºC

Ion source: 240ºC, Transfer line: 240ºC, Ionization mode: Electron ionization

Analysis mode: Scan, Scan rate: 300 msec, Mass range: 30-250

Analyses were performed by H. Mitsunaga (Research and Development Division, Kikkoman Corporation).

Supplemental Table 1. Differences in aspects of palatability for pairings of sashimi and shade wine between sake drinkers and sake non-drinkers

Questions *Descriptive value (1=Not at all, 5=Very much so)*	Score	
Related to reward aspect	Sake drinker (n=47)	Sake non-drinker (n=44)
I think the taste is likely to be addictive for me	3.03±0.15	2.77±0.16
I feel the taste will make me take more	3.32±0.14	2.97±0.15
The taste of this food will make me take another sip	3.48±0.13	3.25±0.17
Related to cultural aspect		
I am used to this taste	3.31±0.15	2.72±0.17*
I have eaten this food many times before	3.51±0.14	3.25±0.17
I have seen this food in advertisements or heard of it before	3.67±0.16	2.93±0.18*
Related to informational aspect		
I think this food looks good	4.01±0.10	3.95±0.14
This food seems expensive	3.82±0.12	3.59±0.15

Values are presented as means ± SEM
* $p<0.05$ between sake drinkers and sake non-drinkers (t-test)
